# Awareness and attitudes towards ear health in classical music students—advancing education and care for professional ear users

**DOI:** 10.3389/fpsyg.2025.1497674

**Published:** 2025-05-26

**Authors:** Moë Fitzlaff, Raphaël Jecker, Alexandra Müller, Mareike Riegert, Cosima Riemenschnitter, Teresa Wenhart, Katrin Bucher, Tobias Kleinjung, Dorothe Veraguth, Horst Hildebrandt, David Bächinger

**Affiliations:** ^1^Department of Otorhinolaryngology, Head and Neck Surgery, University Hospital Zurich, Zurich, Switzerland; ^2^University of Zurich, Zurich, Switzerland; ^3^Department of Music, Zurich University of the Arts, Zurich, Switzerland; ^4^University of Music and Performing Arts Stuttgart, Stuttgart, Germany; ^5^Independent Researcher, Zurich, Switzerland; ^6^Swiss National Accident Insurance Fund (SUVA), Lucerne, Switzerland; ^7^Basel Academy of Music, University of Applied Sciences and Arts Northwestern Switzerland, Basel, Switzerland; ^8^Swiss University Centre for Music Physiology, Zurich, Switzerland; ^9^Swiss University Centre for Music Physiology, Basel, Switzerland

**Keywords:** auditory system, prevention, performing arts medicine, hearing, music education, health education, survey

## Abstract

**Background and aim:**

Classical music students, as a key group of professional ear users (PEUs), rely heavily on their auditory perception, making ear health critical to their education and careers. However, significant gaps in their knowledge of hearing health and protection have been previously identified, while data on non-noise-related risk factors and broader aspects of ear health remain scarce. This study aimed to evaluate classical music students’ knowledge of ear health, including ear anatomy, common ear disorders, and non-noise-related risk factors such as ototoxic medications and cardiovascular risk factors, as well as attitudes toward specialized ear health care. The goal was to inform the development of tailored educational programs and evaluate the need for specialized ear health care in performing arts medicine.

**Methods:**

A questionnaire specifically designed for the purpose of the present study (Professional Ear User Questionnaire) was distributed to classical music students at music schools in Switzerland and Germany, as well as to medical and general students at a Swiss university. Statistical analyses, including Fisher’s exact test and principal component analysis, explored response patterns and identified factors influencing ear health knowledge and behavior.

**Results:**

Data were collected from 209 music students and two control groups of 65 medical students and 40 general students. Significant gaps in ear health knowledge were identified, with only 37.8% familiar with common ear disorders. A total of 52.4% rarely or never used hearing protection, despite 84.4% expressing concerns about potential hearing deterioration. Many were unaware of non-noise-related risk factors, such as ototoxic medications. Only 27.4% knew of an ear specialist for PEUs, yet 72.1% preferred a hearing exam at a specialized clinic.

**Conclusion:**

The study highlights the need for comprehensive ear health education tailored to the unique needs of classical music students and other PEUs. Educational programs should cover both noise and non-noise-related risk factors and promote early hearing protection. The preference for specialized care underscores the importance of establishing dedicated ear health clinics for PEUs. Collaborations between (university) music schools, healthcare providers, and policymakers is crucial to protect the ear health of music students, musicians, and other PEUs, ensuring their ability to perform without preventable ear disorders.

## 1 Introduction

Fully intact hearing is crucial for “Professional Ear Users” (PEUs), such as musicians, instrument makers, sound engineers, and professionals in fields not directly related to music, including sonar technicians or forensic phoneticians (Hall and Santucci, 1995; Bächinger et al., 2022). PEUs place exceptional demands on their ear health and the prevention of ear disorders since their professional activities rely on a highly developed and trained auditory perception (Kishon-Rabin et al., 2001; Bächinger et al., 2022). Among PEUs, classical musicians and music students represent a particularly significant group, which are known to have insufficient knowledge regarding the prevention of noise-induced hearing loss (NIHL) and to use hearing protection less frequently than recommended (Chesky, 2011; Greasley et al., 2020). In music schools’ education programs on performance-related injuries, ear health often receives little attention compared to musculoskeletal, vocal, or mental health (Chesky et al., 2006; Hildebrandt, 2009). Approximately half of music students are exposed to sound levels exceeding the commonly recommended exposure limit of 85 A-weighted decibels averaged over an eight-hour workday (Noise and Hearing Loss, National Institute for Occupational Safety and Health, 2024; Pawlaczyk-łuszczyńska et al., 2021). Since it is estimated that around 50% of NIHL manifests within the first 3 years of exposure to harmful sound, with the remaining hearing loss developing gradually over subsequent decades, early protection is paramount, including protection from leisure noise (Degeest et al., 2022; Dance and Zepidou, 2024). Unsurprisingly, there is evidence of impaired hearing sensitivity among music students, as assessed by objective hearing tests such as otoacoustic emissions (Pawlaczyk-łuszczyńska et al., 2021). Due to excessive sound exposure, (classical) musicians are also at a high risk of hearing loss after completing their studies with an estimated hazard ratio of nearly 4 over 4 years compared to the normal population (Ostri et al., 1989; McBride et al., 1992; Palin, 1994; Schink et al., 2014). Preventing ear and hearing disorders is crucial for PEUs, particularly for (classical) music students and musicians, as even minor auditory disruptions can critically impair their performance abilities (Hall and Santucci, 1995; Bächinger et al., 2022). Furthermore, hearing disorders have been shown to exacerbate performance anxiety, elevate work-related stress, and increase the risk of musculoskeletal performance-related injuries (Narducci, 2020).

Assessing ear health knowledge and attitudes among music students and musicians has primarily focused on noise as the main risk factor and attitudes toward noise protection, including hearing protection use (Chesky et al., 2006; Matei et al., 2018). However, less attention has been given to other risk factors, such as ototoxic drugs or cardiovascular disease risk factors, which are known be associated with worse hearing sensitivity (Gates et al., 1993; Oron et al., 2014). Additionally, attitudes towards potential hearing loss, including anxiety about hearing damage and apprehension regarding hearing assessments, have been underexplored. Moreover, little is known of students’ knowledge about ear symptoms and disorders beyond noise-induced conditions, as well as their awareness and attitudes toward specialized ear health clinics. These knowledge gaps likely contribute to preventable hearing damage, hinder access to specialized ENT practices specialized in treating PEUs, and may lead to significant psychosocial stress, consecutive performance-related injuries, and reduced work ability (Obrien et al., 2014; Narducci, 2020). Yet, it is important that music students are educated early and frequently about ear health, starting during their early education (Chesky, 2011).

In this study, we aimed to evaluate the knowledge of music students regarding ear physiology, hearing, and the prevention and treatment of ear disorders. We hypothesized that music students, as an important group of PEUs, are not adequately informed in these areas. The data collected could be used to design tailored educational programs and curricula, establish specialized ear health clinics, and enhance collaborative efforts between music schools and specialized ENT physicians.

## 2 Materials and methods

### 2.1 Participants

Only anonymized data was collected, with no personally identifiable information. Consequently, formal ethical approval was waived by the local Ethics Committee. Nonetheless, all participants provided informed consent for the use of their data for scientific purposes by ticking a box on the questionnaire. The questionnaire was offered to classical music performance students during courses and lectures at three university music schools between October 2023 and May 2024: Department of Music at the Zurich University of the Arts (Zurich, Switzerland), Basel Academy of Music at the University of Applied Sciences and Arts Northwestern (Basel, Switzerland) and the University of Music and Performing Arts Stuttgart (Stuttgart, Germany). The inclusion criteria required respondents to be adults and classical music performance students. Other students, such as composition or music pedagogy students were excluded. Additionally, they were required to provide consent for the use of their data for scientific purposes. As a comparison group, and with the aim of assessing a diverse sample of students including different levels of knowledge, medical and general students were recruited through personal contacts and advertisements at the University of Zurich.

### 2.2 Professional ear user questionnaire (PEU-Q)

The Professional Ear User Questionnaire (PEU-Q) is a novel questionnaire designed for the present study to assess knowledge and beliefs about ear health. It also includes questions about respondents’ ear symptoms and their attitudes toward medical resources. The PEU-Q was developed based on the opinions and suggestions of performing arts medicine specialists, ear physicians, as well as musicians and PEU patients. This group of experts and patients also participated in a pilot test to assess face validity (*n* = 10) to evaluate the clarity of instructions, wording, and overall comprehensibility of the PEU-Q. Feedback from the pilot was used to refine the questionnaire. The PEU-Q has not been fully validated psychometrically, as it was not designed to assess a unified construct. A translated version is presented in Table 1. The original questionnaire is provided as Supplementary material S1.

**Table 1:** Items of the professional ear user questionnaire.

I have a good understanding of how hearing works.
I am familiar with the most important and common ear diseases.
I know how to effectively protect my hearing.
I am aware of the situations in which I need to protect my hearing.
I experience temporary or transient problems with my hearing, such as hearing loss, distorted hearing, unpleasantly increased hearing or ringing in the ears (tinnitus).
I have problems with my ears (pain, hearing loss, dizziness, ringing in the ears) in connection with air and/or car travel.
I am concerned about the potential deterioration of my hearing over the course of my life.
I am aware that sound can be harmful to the ear depending on both its volume and exposure time.
I use hearing protection (e.g., foam ear plugs, otoplastics).
The idea of using hearing protection triggers negative feelings (e.g., discomfort, feelings of shame or stress).
I am exposed to noise in my leisure time (e.g., music clubs, hobbies).
I am aware that medications can damage my hearing.
I make sure room acoustics are gentle on my hearing when practicing or working (e.g., sound-absorbing wall elements, carpets, sufficiently large rooms).
I smoke (more than 3 times/week) or drink alcohol regularly (more than 3 times/week).
I clean my ear canal with cotton buds or other instruments.
I have taken any of the following medications in the last 12 months: Aspirin, Alka-Seltzer, Aspégic, or Aspro.
The idea of having my hearing health checked makes me feel uncomfortable.
I am afraid that my hearing may already be damaged, but I am avoiding medical examination.
I would feel happier about having a medical hearing examination if it was at a clinic specialized in ear health for Professional Ear Users.
I would seek advice of an ear specialist for Professional Ear Users through individual consultations regarding risk factors, prevention and screening.
I know an ear specialist (e.g., at an ENT practice or clinic) who specializes in treating Professional Ear Users, whom I could contact or be referred to if I experience ear problems.
I have visited an ear specialist in the last 12 months.
I have had a hearing test in the past with an audiologist, or at an ENT specialist.
I have had negative experiences with ear specialists in the past (e.g., insufficient consideration/appreciation of complaints, especially related to professional activity).

Responses are recorded on a 4-point Likert scale (strongly disagree, disagree, agree, strongly agree or never, rarely, often, very often).

### 2.3 Statistical analysis and data reporting

All statistical tests were selected before data collection. If not otherwise specified, values are reported as absolute number and percentage. To compare the distribution of answers between groups, a Fisher’s exact test was performed. A *p*-value < 0.05 was considered as statistically significant.

A principal component analysis (PCA) was conducted on the responses to further explore the underlying structure of the data. The PCA employed Varimax rotation with Kaiser normalization to maximize the variance of factor loadings. Varimax rotation helps to simplify the components by making high loadings higher and low loadings lower for each factor. This rotation method was chosen to enhance the interpretability of the factors by minimizing the number of variables that have high loadings on each factor. A cut-off value of 0.40 was used for factor loadings (Stevens, 2001). The number of factors to be extracted was determined using Kaiser’s criterion, which retains factors with eigenvalues greater than 1, as they account for more variance than a single observed variable.

Completed questionnaires were transferred into an Excel sheet and reviewed for accuracy by two independent researchers. Incomplete responses with missing data were excluded. No extreme outliers were identified requiring exclusion or transformation. No data transformations were applied, as all variables were analyzed in their original categorical or ordinal form. The cleaned dataset was then imported into IBM SPSS Statistics, version 29 (IBM Corp., Armonk, NY, USA) and Prism for MacOS, version 10.2.3 (GraphPad Software, Inc., La Jolla, CA, USA), where statistical analyses were performed and graphs were generated. Data is reported according to the Consensus-Based Checklist for Reporting of Survey Studies (CROSS) guidelines (Sharma et al., 2021).

## 3 Results

### 3.1 Participants and demographics

A total of 209 classical music students, 65 advanced medical students (in the last 3 years of medical school), and 40 general students from various subjects (“general students”; law, psychology, biomedicine) completed the PEU-Q. The three groups did not exhibit significantly different gender distribution with 51.2% females in the music students, 61.5% in medical students and 57.5% in general students. The majority of participants across all groups were aged 18–25 years, with 88.5% of music students, 79.2% of medical students, and 75.0% of general students falling into this category.

### 3.2 Response distribution

**Figure 1:**
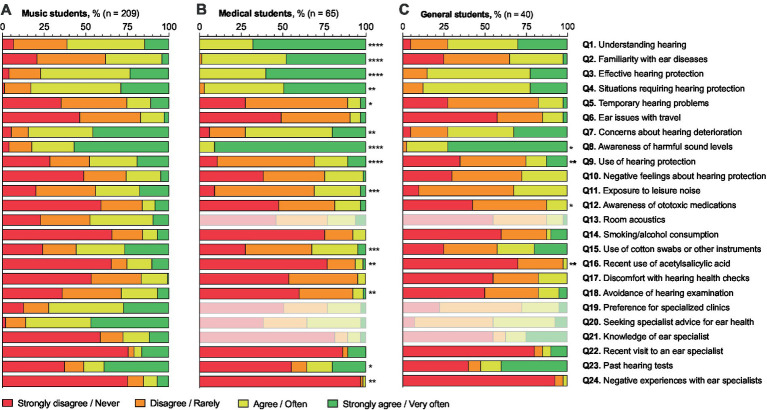
Answer distribution in the professional ear user questionnaire of music students (*n* = 209; **A**), medical students (*n* = 65; **B**), and general students (*n* = 40; **C**). Answer distributions to questions irrelevant or not applicable to medical and general students are shown in light colors (Q13, Q19–22). The figure includes a statistical comparison of the answer distributions of medical and general students compared to music students, with significance levels indicated by asterisks (*, *p* < 0.05; **, *p* < 0.01; ***, *p* < 0.001; ****, *p* < 0.0001). The x-axis represents the percentage of students in each response category: “Strongly disagree/Never,” “Disagree/Rarely,” “Agree/Often,” and “Strongly agree/Very often.”

The distribution of responses to the PEU-Q for each group, as well as the inter-group comparisons of the response distribution for each question is illustrated in Figure 1. Furthermore, to facilitate interpretation of the analysis, responses were dichotomized into binary categories, consolidating answers into (strongly) disagree/never/rarely or (strongly) agree/(very) often. Among the music students, 61.0% felt they had a good understanding of how hearing works (question [Q] 1), but only 37.8% felt familiar with the most important ear disorders (Q2). A majority felt educated on how (76.9%) and when (82.9%) to protect hearing (Q3–4). Temporary symptoms, such as hearing loss, dysacusis, hyperacusis, or tinnitus, were experienced by 25.2% (Q5). A total of 84.4% of music students were concerned about potential hearing deterioration over their lifetime (Q7). Hearing protection was used at least often by 47.6% (Q9), with 25.5% feeling that it can trigger negative feelings (Q10). Attention to room acoustics while practicing was indicated by 47.3% (Q13).

Risk factors for hearing health in music students include noise during leisure time, i.e., noise exposure unrelated to the students’ academic activities or instrumental practice (43.9%; Q11), medication, and cardiovascular disease risk factors. Only 15.9% were aware that medications can damage hearing (Q12), with 25.0% having taken acetylsalicylic acid in the past 12 months (Q16). Additionally, 15.8% of students smoked or drank alcohol regularly (more than 3 times/week; Q14). Cotton buds or other instruments to clean the external auditory canal were used by 55.6% (Q15).

The idea of having their hearing health checked made 16.4% uncomfortable (Q17), and 28.6% were afraid their hearing might already be damaged (Q18). A total of 51.0% rarely or never had their hearing tested (Q23), while 20.9% visited an ear specialist in the last 12 months (Q22). Negative experiences with ear specialists were reported by 15.1% (Q24). Preferred examination at a clinic specialized in ear health for musicians/PEUs was indicated by 72.1% (Q19), and 85.8% would seek advice from an ENT physician specializing in musicians/PEUs for preventive or screening measures (Q20). A total of 27.4% knew an ear specialist who specializes in treating PEUs (Q21).

Response distributions were compared between music student and the other student groups. Compared to music students, significantly different distribution patterns were found in medical students for Q1–5, Q7-9, Q11, Q15-16, Q18 and Q23–24. In other words, medical students felt better informed about how hearing works, risk factors such as noise or acetylsalicylic acid, and where they need to apply protective measures. On the other hand, they expressed less concern about potential hearing deterioration over their lifetime and had experienced fewer negative encounters with an ear physician. In general students, significantly different distribution patterns were found for Q8–9, Q12, Q18, and Q16, indicating that general students had a higher awareness of harmful sound levels but used hearing protection less frequently. Furthermore, although they were less aware of ototoxic medications, general students used acetylsalicylic acid less often.

### 3.3 Principal component analysis

**Figure 2:**
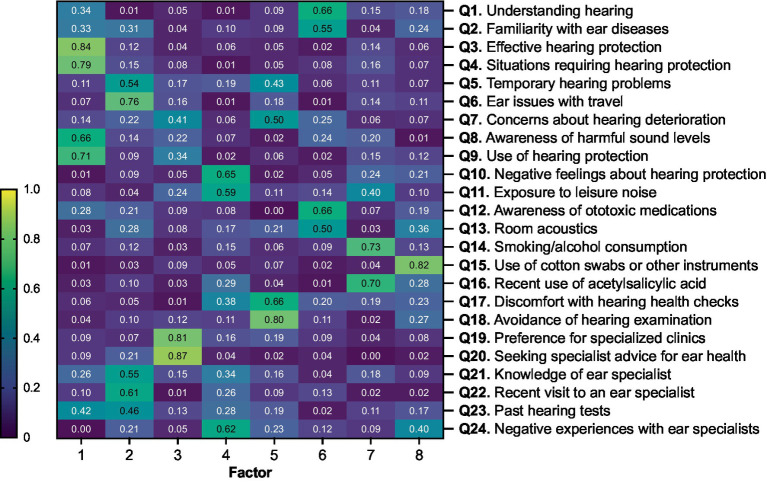
Principal component analysis (PCA) of responses from music students (*n* = 209) to the professional ear user questionnaire. The component matrix displays the factor loadings for each question, indicating the degree to which each question (Q) correlates with each factor. A higher absolute value of a loading indicates a stronger association between the item and the factor. The heatmap color scale ranges from 0 (low correlation, dark blue) to 1 (high correlation, yellow).

A PCA was conducted to explore the underlying structure of the responses of the music students’ group (Figure 2). The exploratory PCA aimed to identify the key factors that account for the variability in the data. The analysis revealed eight factors, with loadings > 0.40 on specific questions.

Factor 1 covered awareness and practices of hearing protection, including respondents’ knowledge and behaviors related to protecting their hearing. High loadings from Q3 (0.84), Q4 (0.79), Q9 (0.71), and Q8 (0.66) indicate that respondents with higher awareness and proactive behaviors regarding hearing protection tend to understand when and how to use protective measures effectively. Factor 2 included questions on ear symptoms and medical resources, with high loadings from Q5 (0.54), Q6 (0.76), Q21 (0.55), Q22 (0.61), and Q23 (0.46). This suggests that the presence of ear symptoms, such as temporary hearing problems and issues during travel, correlates with visits to ENT doctors, knowledge of ear disorders, and the likelihood of having undergone hearing tests. Factor 3 was characterized by the seeking of specialist care, with high loadings from Q19 (0.81) and Q20 (0.87). This factor reflected that concerns about the potential deterioration of hearing over their lifetime correlated with the respondents’ preference for specialized care. Factor 4, which includes high loadings from Q10 (0.65) and Q11 (0.59) on negative perceptions and experiences, suggests a distinct cluster of negative perceptions and experiences related to hearing protection and ear specialists. With the highest loadings from Q17 (0.66) and Q18 (0.80), Factor 5 showed a close relationship between discomfort with hearing health checks and avoidance of medical examinations due to fear of discovering hearing damage. It also includes concerns about potential hearing deterioration. Factor 6 captured knowledge of ear health and awareness of associated risk factors, with high loadings from Q1 (0.66) and Q12 (0.66). This suggests that respondents who are knowledgeable about how hearing works are also aware of ototoxic medications and the importance of favorable room acoustics. Factor 7 was defined by high loadings from Q14 (0.73) and Q16 (0.70). This factor indicates a common construct related to risky behaviors, including the consumption of substances like alcohol and smoking, along with the use of acetylsalicylic acid.

## 4 Discussion

This study reveals significant gaps in classical music students’ knowledge and attitudes toward ear health, indicating areas for improvement. While many students are aware of noise-related risks and the need for hearing protection, fewer than 50% regularly use hearing protection. Alarmingly, a substantial number of students experience ear symptoms like tinnitus and hyperacusis, with limited awareness of ear disorders and non-noise-related risk factors. Our results underscore the need for a more nuanced and comprehensive ear health education and care tailored to the unique needs of classical music students and PEUs in general.

### 4.1 Understanding of hearing and ear disorders

Around 60% of music students indicated that they have a reasonable understanding of how hearing functions, but only about 40% were familiar with common signs and symptoms of ear-related disorders. These results were similar to those of general students, but significantly different from those of medical students, who expectedly demonstrated a higher level of knowledge. Understanding the physiology and pathophysiology of hearing is crucial for comprehending ear health and implementing effective preventive and therapeutic measures. This knowledge gap among music students should be addressed as early as possible to foster proactive ear health practices and acceptance of hearing protection methods. Schools and educational programs should prioritize integrating detailed ear health curricula that cover a wide range of topics from basic auditory function to ear conditions and their prevention.

### 4.2 Hearing protective behavior

Exposure to non-amplified music, including individual practice with various string, woodwind, or brass instruments, is a well-documented cause of NIHL (sometimes called “music-induced hearing loss” in musicians), yet hearing protection is underutilized (Greasley et al., 2020; SUVA, 2021). Our study shows that while a comparable portion of all the students investigated understand when and how to protect their hearing, actual use of protection is low, with 52% of music students rarely or never using it. Despite progress in awareness about hearing protection, the trend shown in the present study is consistent with the previously reported 49 to 62% (Lonsdale and Boon, 2016; Olson et al., 2016) suggesting that knowledge alone does not necessarily translate into protective behavior (Miller et al., 2007; Laitinen and Poulsen, 2008; Zander et al., 2008). Still, our principal component analysis suggests that protective behavior is related to knowledge about hearing health. These and previous findings highlight the need for proactive educational strategies and supportive measures (e.g., financial support) to encourage early adoption of hearing protection (Richter et al., 2011). This could also mitigate the stigmatizing effect of hearing protection, as found by our study and others, with more than half of the music students indicating that hearing protection triggers negative feelings in themselves (Laitinen and Poulsen, 2006; Mina et al., 2023).

### 4.3 Room acoustics and noise exposure during practice

When educating PEUs about hearing health, often neglected topics include noise exposure during individual practice as well as non-noise-related risk factors (Hildebrandt, 2009; Rodrigues et al., 2015; Matei et al., 2018). Studies have shown that during individual practice sessions, music students are exposed to sound levels well above the recommended exposure limits, often without using hearing protection (Rodrigues et al., 2019). This is corroborated by our findings, revealing that only around half of the music students ensure optimal room acoustics during practice. While the question may underestimate the impact of institutional measures already implemented to improve room acoustics, our results reflect a broader trend in which room acoustics during individual practice are often overlooked. This is particularly evident in comparison to the extensive attention given to concert halls and opera houses, particularly regarding the reduction of hazardous noise levels for musicians (Dance and Lorenzetto, 2009; Dance et al., 2010). Given that individual practice typically accounts for the most significant portion of a musician’s sound exposure, and often occurs in small rooms with poor acoustics and insufficient hearing protection (Behar et al., 2006; Dance and Lorenzetto, 2009; Dance et al., 2010), it is crucial to address room acoustics during practice to mitigate the risk of hearing damage.

### 4.4 Recreational noise exposure

Another neglected risk factor in PEUs is the exposure to recreational noise (Clark, 1991). While it has been emphasized that managing noise dosage requires addressing both occupational and recreational exposures, there is limited data available on this topic (Olson et al., 2016). As an example, a study on a small sample of sound engineers revealed that a significant portion were exposed to recreational noise, such as attending music events or using power tools (Ntlhakana and Heliopoulos, 2020). In our cohort, around 80% of music students indicated a significant noise exposure in their leisure time. It is therefore important to educate PEUs that reducing overall noise dosage includes also reducing recreational exposure (Rodrigues et al., 2015; Olson et al., 2016).

### 4.5 Non-noise-related risk factors

Additionally, our study highlights that music students are frequently exposed to non-noise-related risk factors, such as smoking and alcohol consumption, which are known to exacerbate hearing loss (Cruickshanks et al., 1998; Upile et al., 2007). Furthermore, an alarming 80% of students were unaware of the ototoxic potential of medications such as acetylsalicylic acid, which is used by a third of the students surveyed. We observed a positive correlation between various individual risk factors (e.g., noise, ototoxic medications, smoking), which, though minor on their own, may be significant for PEUs due to their above-average hearing abilities. Importantly, these factors can cause exponential damage when combined, particularly together with noise exposure (McFadden and Plattsmier, 1983; Durrant et al., 2009). Lastly, hearing health education should address proper ear canal cleaning, as 75% of music students in our study use cotton buds, which are not recommended due to the canal’s self-cleaning ability and the risk of injury (Kravitz et al., 1974).

### 4.6 Emotional and psychological aspects of hearing health

In our study, over 70% of music students avoided medical examinations and hearing tests due to fear of existing hearing damage. This avoidance was linked to concerns about long-term hearing deterioration and discomfort with hearing tests, and it correlated with temporary hearing symptoms reported by about two-thirds of the students. Combined with the previously mentioned negative feelings triggered by hearing protection, these findings underscore the emotional and psychological aspects tied to ear and hearing health (Hall and Santucci, 1995). Similarly, reported hearing deficits in musicians are well-known to correlate with psychological symptoms, impaired social environments, and increased stress (Laitinen and Poulsen, 2008; Hasson et al., 2009; Narducci, 2020).

### 4.7 Importance of regular hearing assessments

A significantly larger portion of music students had their hearing tested compared to medical students (63% vs. 45%). Although higher than previous reports of 28 to 41% for music students (Rodrigues et al., 2015; Lonsdale and Boon, 2016) and 33% for professional musicians (Greasley et al., 2020), we recommend that all students should undergo hearing tests, especially when entering university music schools. The Royal Academy of Music in London has implemented a program of regular hearing tests, which could serve as a model for other institutions (Dance and Zepidou, 2024). Such tests can provide a baseline for future assessments and facilitate the early detection of hearing loss (Olson et al., 2016; Dance and Zepidou, 2024).

### 4.8 Improving educational programs and curricula

To address ear health challenges among PEUs, especially classical music students, educational programs should integrate comprehensive ear health education early into music curricula, including basic knowledge on hearing, occupational noise, and non-noise-related risk factors (Laitinen and Poulsen, 2008; Hildebrandt, 2009; Zepidou and Dance, 2010). The American National Association of Schools of Music recommends providing students with “basic information regarding the maintenance of hearing” (National Association of Schools of Music Handbook 2023-24, 2024). However, given current and past findings, these programs should be broadened to include not only basic information about hearing and occupational noise protection, but also information on recreational noise, non-noise related risk factors, and ear disorders. Education from credible sources, like ENT physicians, is crucial to increase relevance and foster a supportive culture (Mina et al., 2023). Additionally, ear health curricula may address psychological aspects such as risk perception and conviction as well as learning strategies for managing emotions. These aspects, combined with leveraging social influence, including through role models such as teachers, can enhance hearing protection acceptance, reduce negative perceptions, and ensure students know when and where to seek help, ultimately safeguarding their ear health and professional careers (Chesky et al., 2006; Griest et al., 2007; Mina et al., 2023).

### 4.9 The value of specialized ear health clinics

PEUs, especially music students, need to know not only when but also where to seek help for ear problems (Chesky et al., 2006). Our study found that only a quarter of music students were aware of an ear specialist for PEUs, i.e., a performing arts medicine expert in ear health. Yet, 72% would prefer a hearing exam at a specialized clinic, and 86% would seek advice on risk factors, prevention, and screening from such clinics. This emphasizes the need to connect music students with specialized ear physicians. Previous studies show that musicians often do not seek help due to lack of awareness or stigma (Greasley et al., 2020). Early contact with specialized professionals can facilitate education, screening, and awareness, making collaboration between university music schools and ENT specialists essential.

### 4.10 Limitations

This study has several limitations. First, the PEU-Q was specifically developed for this study and underwent pilot testing for face validity, however, it lacks full psychometric validation, which could be the focus of future studies. Second, while the sample includes students from various fields, it may not fully represent all classical music students or PEUs, as demographic, regional characteristics and differing prior knowledge might limit the generalizability. Moreover, slightly differing questionnaire distribution methods could introduce bias and affect the representativeness of the different student samples. Third, the use of self-reported questionnaires could introduce biases like social desirability and recall bias, leading to potential over- or underestimation of participants’ knowledge, attitudes, and behaviors regarding ear health. Additionally, as hearing loss assessment was not a focus of this study, the findings rely solely on self-reported data as no audiometric data are available, which may limit objectivity of the results. Finally, the cross-sectional design provides a snapshot in time, but does not track changes over time. Longitudinal studies would be valuable to observe how knowledge, attitudes, and behaviors evolve with ongoing education and awareness efforts.

## 5 Conclusion

This study reveals significant knowledge gaps and attitudes towards ear health among classical music students. Despite the vital role of intact hearing for PEUs, many students lack comprehensive knowledge about ear physiology, disorders, and hearing protection. Educational programs should address not only noise protection but also non-noise-related risk factors, ear disorders, and proper ear cleaning practices, aspects that are currently being integrated into our local health education program. The preference for specialized care indicates the need for dedicated ear health clinics for PEUs, ideally within performing arts medicine centers. A collaborative effort between music university music schools, healthcare providers, and policymakers is crucial to develop comprehensive ear health education, and resources tailored to music students. By fostering awareness and proactive hearing care, we can protect the hearing health of future generations of musicians and other PEUs, ensuring their ability to excel in their professions without preventable hearing damage.

## Data Availability

The datasets generated and analyzed during the current study are available from the corresponding author on reasonable request.
