# Supplementary material for: Awareness and attitudes towards ear health in classical music students—advancing education and care for professional ear users
Source: Front Psychol. 2025 May 26;16:1497674. doi: 10.3389/fpsyg.2025.1497674 (PMC12146397; doi:10.3389/fpsyg.2025.1497674)
Supplement: Supplementary file 1 [file Data_Sheet_1.pdf]

**Professional Ear User Questionnaire (PEU-Q)**	⊖ ⟵ Überhaupt nicht zutreffend	Eher nicht zutreffend	Eher zutreffend	⟶ ⊕ Voll zutreffend
	Nie	Selten	Oft	Sehr oft
**1.** Ich habe eine gute Vorstellung davon, wie das Hörorgan funktioniert.	☐	☐	☐	☐
**2.** Ich kenne die wichtigsten und häufigsten Ohrerkrankungen.	☐	☐	☐	☐
**3.** Ich weiss, wie ich mein Gehör schützen kann.	☐	☐	☐	☐
**4.** Ich weiss, in welchen Situationen ich mein Gehör schützen muss.	☐	☐	☐	☐
**5.** Ich habe kurzzeitige bzw. vorübergehende Probleme mit meinem Gehör, z. B. eine Hörminderung, verzerrtes Hören, unangenehm gesteigertes Hörvermögen oder ein Ohrgeräusch (Tinnitus).	☐	☐	☐	☐
**6.** Ich habe Probleme mit dem Ohr (Schmerzen, Hörminderung, Schwindel, Ohrgeräusch) im Zusammenhang mit Flug- und/oder Autoreisen.	☐	☐	☐	☐
**7.** Ich habe Angst, dass mein Gehör im Verlauf des Lebens schlechter werden könnte.	☐	☐	☐	☐
**8.** Ich bin mir bewusst, dass die Schädlichkeit von Schall sowohl durch die Lautstärke als auch die Expositionszeit beeinflusst wird.	☐	☐	☐	☐
**9.** Ich wende einen Gehörschutz an (z. B. Oropax, otoplastischer Gehörschutz).	☐	☐	☐	☐
**10.** Die Anwendung eines Gehörschutzes ist für mich mit negativen Gefühlen verbunden (z. B. Unwohlsein, Scham- oder Stressgefühl).	☐	☐	☐	☐
**11.** Ich bin in meiner Freizeit lautem Schall ausgesetzt (z. B. Musikclubs, Hobbys).	☐	☐	☐	☐
**12.** Ich bin mir bewusst, dass es Medikamente gibt, die das Gehör schädigen.	☐	☐	☐	☐
**13.** Ich achte mich auf eine gehörschonende Raumakustik beim Üben/Arbeiten (z. B. schalldämmende Wandelemente, Teppiche, genug grosse Räume).	☐	☐	☐	☐
**14.** Ich rauche (mehr als 3-mal/Woche) oder trinke regelmässig Alkohol (mehr als 3-mal/Woche).	☐	☐	☐	☐
**15.** Ich reinige meinen Gehörgang mit Wattestäbchen oder sonstigen Instrumenten.	☐	☐	☐	☐
**16.** Ich habe in den letzten 12 Monaten eines der folgenden Medikamente eingenommen: Aspirin, Alka-Seltzer, Aspégic.	☐	☐	☐	☐
**17.** Der Gedanke, mich bezüglich meiner Gehörgesundheit untersuchen zu lassen, bereitet mir Unwohlsein.	☐	☐	☐	☐

**Professional Ear User Questionnaire (PEU-Q)**<br>*(Fortsetzung)*	⊖ Überhaupt nicht zutreffend<br>Nie	Eher nicht zutreffend<br>Selten	Eher zutreffend<br>Oft	⊕ Voll zutreffend<br>Sehr oft
**18.** Ich habe Angst davor, dass mein Gehör bereits geschädigt sein könnte, vermeide aber eine medizinische Untersuchung.	☐	☐	☐	☐
**19.** Das Vorhandensein einer spezialisierten ohrenärztlichen Anlaufstelle für Professional Ear Users würde meine Hemmschwelle für eine medizinische Untersuchung des Gehörs senken.	☐	☐	☐	☐
**20.** Ich würde eine spezialisierte ohrenärztliche Anlaufstelle für Professional Ear Users auch für eine Beratung in Anspruch nehmen (individuelles Gespräch und Informationen bezüglich Risikofaktoren, Prävention und Vorsorgeuntersuchungen).	☐	☐	☐	☐
**21.** Ich kenne eine auf Professional Ear Users spezialisierte ohrenärztliche Anlaufstelle, an die ich mich bei Ohrenproblemen wenden könnte (z. B. HNO-Praxis oder HNO-Klinik).	☐	☐	☐	☐
**22.** Ich war in den letzten 12 Monaten bei einem Ohrenarzt.	☐	☐	☐	☐
**23.** Ich habe mein Gehör in der Vergangenheit mit einem professionellen Hörtest testen lassen.	☐	☐	☐	☐
**24.** Ich habe in der Vergangenheit negative Erfahrungen mit Ohrenärzt:innen gemacht (z. B. keine ausreichende Berücksichtigung/Würdigung der Beschwerden, speziell im Zusammenhang mit der beruflichen Tätigkeit).	☐	☐	☐	☐

**Persönliche Angaben**						
Alter:	☐ ≤ 18 Jahre	☐ 18–25 Jahre	☐ 26–30 Jahre	☐ 31–40 Jahre	☐ 41–50 Jahre	☐ > 51 Jahre
Geschlecht:	☐ männlich	☐ weiblich	☐ divers	☐ keine Angabe		
Beruf/Ausbildung zu:	☐ Musiker:in	☐ Musikwissen-schaftler:in	☐ Ton-meister:in	☐ Instrumenten-bauer:in	☐ anderes: ____________	
Ich bin damit einverstanden, dass meine Daten anonymisiert zu wissenschaftlichen Zwecken verwendet werden.	☐ Ja	☐ Nein				
Datum:						

Vielen Dank!
